# Supplementary material for: White plague among the “forgotten people” from the Barbaricum of the Carpathian Basin–Cases with tuberculosis from the Sarmatian-period (3rd–4th centuries CE) archaeological site of Hódmezővásárhely–Kenyere-ér, Bereczki-tanya (Hungary)
Source: PLoS One. 2024 Jan 10;19(1):e0294762. doi: 10.1371/journal.pone.0294762 (PMC10781108; doi:10.1371/journal.pone.0294762)
Supplement: S1 Text — (PDF) [file pone.0294762.s001.pdf]

S1 Text: The Sarmatian period (1st–5th centuries CE) in the Barbaricum of the Carpathian Basin.

The history of Sarmatians in the Carpathian Basin started in the middle of the 1st century CE, when the political and ethnic image of this geographical region underwent fundamental changes. The Roman administrative system was gradually established in the Danube region (province of Pannonia), the first Germanic-speaking barbarian tribes (e.g., Quadi and Marcomanni) appeared to the north, while some of the Iranian-speaking Sarmatian groups took advantage of the temporary weakening of the Dacian Kingdom and moved into the northern part of the Danube-Tisza Interfluve. From the 2nd–3rd centuries BCE, the Sarmatian tribes (e.g., Aors, Siraci, Iazyges, and Roxolani) were the dominant nomadic groups in the Eastern European steppe region; from social, economic, and cultural points of view, they were closely linked to the Scythian populations, who ruled the steppe region before them [1-2]. According to written sources, the Iazyges were the first Sarmatian tribes, who moved into the Carpathian Basin from the Lower Danube region [3]. By the end of the 1st century CE, the Sarmatians already became involved in political and diplomatic events – as enemies of the Roman Empire, they were permanent participants in wars that were led by Emperor Domitian against the local barbarian tribes. During the so-called Marcomannic-Sarmatian Wars, which broke out in the mid-2nd century CE, the Sarmatians led several campaigns against the Danubian and Balkan provinces of the Roman Empire [4-6]. In the decades following this war, their role in the region became increasingly important, as reflected in the high influx of Roman goods into their territories [7-8]. Presumably during the 2nd–4th centuries CE, Sarmatian groups from the east continued to arrive to the eastern part of the Carpathian Basin (Barbaricum); their typical oriental burial customs and artefacts can be traced in the archaeological record for a generation [9-10]. The Sarmatians joined forces with various Germanic tribes (e.g., Quadi and Vandali) to

attack and sack the province of Pannonia on several occasions in the 3rd and 4th centuries CE. During the 4th century CE, time and time again, they were presumably dependent on the Roman emperors, who frequently interfered in their internal affairs (development of vassal status) [11-12]. The Sarmatian population survived the Hun period (last quarter of the 4th century CE–second half of the 5th century CE); even after that, they were mentioned by written sources as an ethno-political group in their own right [13-15]. Nonetheless, the Gepidic Kingdom, which took shape in the second half of the 5th century CE, finally integrated and assimilated the Sarmatians by the beginning of the 6th century CE [16].

The Sarmatian groups arriving from the steppes to the Carpathian Basin adopted to the local conditions and abandoned their nomadic way of life relatively quickly, as evidenced by the continuously developing, extensive settlement network and the archaeological finds, which indicate an intensive agricultural activity and craftsman industry [17-19]. Specific ritual elements of the Sarmatian population of the Barbaricum are linked to the steppe traditions in several ways (e.g., ditched graves, burial mounds, and clay incense burners); they changed little over the centuries. On the other hand, their material culture adopted and integrated local features in many aspects (e.g., costume, weapons, ceramics, and Celtic, Dacian, and Roman influences) [7,20-22]. During and after the Marcomannic-Sarmatian Wars, a closer relationship between Sarmatian and Germanic peoples developed, especially in the border zone, in the northern part of the Great Hungarian Plain [23-25]. Nevertheless, more recently there is increasing evidence of close military alliances in the southern part of the Sarmatian territories, as well (e.g., the grave of **HK309** from the archaeological site of Hódmezővásárhely–Kenyere-ér, Bereczki-tanya). As for their burial customs, the Sarmatians practiced an inhumation rite, with the grave pits being usually south-north oriented [26-27]. In many cases, there are signs of post-depositional disturbance of the burials (e.g., contemporary grave robbery). Interestingly, human skeletons were found not only in cemeteries but also in Sarmatian settlement objects (e.g.,

corpses that could have been thrown into storage or garbage pits). In the archaeological literature, there are several explanations for this rare but not unusual phenomenon [28] – they can be imprints of violent events [29], sacrifice or ritual acts [30].

REFERENCES:

1. Melyukova AI, Crookenden J. The Scythians and Sarmatians. In: Sinor D, editor. The Cambridge history of early Inner Asia. Cambridge, UK: Cambridge University Press; 1990. pp. 97–117. doi: 10.1017/CHOL9780521243049.005
2. Kuz'mina EE. The origin of the Indo-Iranians (Leiden Indo-European etymological dictionary series, Volume 3). Leiden, The Netherlands: Brill; 2007.
3. Istvánovits E, Kulcsár V. Az első szarmaták az Alföldön (Gondolatok a Kárpátmedencei jazig foglalásról). [The first Sarmatians in the Great Hungarian Plain (Some notes on the Jazygian immigration into the Carpathian Basin.)]. A Nyíregyházi Jósa András Múzeum Évkönyve 2006;48: 203–237.
4. Dio Cocceianus C, Cary E, Foster HB. Dio's Roman history, with an English translation by Earnest Cary, Ph.D., on the basis of the version of Herbert Baldwin Foster, Ph.D. Cambridge, MA, USA: Harvard University Press & London, UK: William Heinemann Ltd.; 1914.
5. Kovács P. Marcus Aurelius's rain miracle and the Marcomannic Wars. Leiden: The Netherlands: Brill; 2009.
6. Gabler D. Die archäologischen Evidenzen der Markomannisch-Sarmatischen Kriege (166–180 N. Chr.) in den Donauprovinzen [Archaeological evidences on Marcomannic-Sarmatian Wars (AD 166–180) in the Danubian Provinces]. Štud Zvesti 2017;61: 21–40.

7. Vaday A. Kereskedelem és gazdasági kapcsolatok a szarmaták és a rómaiak között. In: Havassy P, editor. Jazigok, roxolánok, alánok. Szarmaták az Alföldön. Gyulai katalógusok 6. Gyula, Hungary: Erkel Ferenc Museum; 1998. pp. 117–144.
8. Bene Z, Istvánovits E, Kulcsár V. Some characteristic types of Roman imports in Sarmatian Barbaricum in the Carpathian Basin (caskets decorated with metal mounts, bronze vessels, mirrors). In: Voß H-U, Müller-Scheeßel N, editors. Archäologie zwischen Römern und Barbaren: zur Datierung und Verbreitung römischer Metallarbeiten des 2. und 3. Jahrhunderts n. Chr. im Reich und im Barbaricum – ausgewählte Beispiele (Gefäße, Fibeln, Bestandteile militärischer Ausrüstung, Kleingerät, Münzen): Internationales Kolloquium, Frankfurt am Main, 19.–22. März 2009. Kolloquien zur Vor- und Frühgeschichte 22. Bonn, Germany: Dr. Rudolf Habelt GmbH; 2016. pp. 743–760.
9. Kulcsár V. Újabb szempontok a hévízgyörki szarmata sírok etnikai meghatározásához. In: Asztalos T, editor. Egy múzeum szolgálatában. Tanulmányok Asztalos István tiszteletére. Múzeumi Füzetek 48. Aszód, Hungary: Osváth Gedeon Museum Foundation; 1998. pp. 75–84.
10. Istvánovits E, Kulcsár V. Sarmatians: History and archaeology of a Forgotten People. Mainz, Germany: Verlag des Römisch-Germanischen Zentralmuseums; 2017. pp. 258–307.
11. Marcellinus A, Rolfe JC. Ammianus Marcellinus, with an English translation by John C. Rolfe, Ph.D, Litt. D. London, UK: William Heinemann Ltd. & Cambridge, MA, USA: Harvard University Press; 1935.
12. Vaday A. Military system of the Sarmatians. In: Istvánovits E, Kulcsár V, editors. International connections of the Barbarians of the Carpathian Basin in the 1st–5th centuries A. D. Nyíregyháza, Hungary: Nyíregyházi Jósa András Museum & Aszód, Hungary: Osváth Gedeon Museum Foundation; 2001. pp. 171–193.

13. Jordanes. Getica (De summa temporum vel origine actibusquegentis Gothorum). In: Mommsen T, editor. Monumenta Germaniae Historica. Auctores Antiquissimi Tomus 5. Berlin, Germany; 1882. pp. 53–138.
14. Vaday A. Late Sarmatian graves and their connections within the Great Hungarian Plain. Slov Archeol. 1994;42(1): 105–124.
15. Masek Z. Settlement research of the 5th century in the core of the Hunnic Empire: A chronological and stylistic approach. In: Rácz Z, Szenthe G, editors. Attila's Europe? Structural transformation and strategies of success in the European Hun period. Budapest, Hungary: Hungarian National Museum; 2021. pp. 361–388.
16. Kiss PA. Who would leave Asia, Africa or Italy to go to Germania? Germanic tribes in the Carpathian Basin. Budapest, Hungary: Martin Opitz Kiadó; 2023. pp. 197–199.
17. Vaday A. Adatok a szarmaták fémművességéhez. A Szántó Kovács János Múzeum Évkönyve 2005;7: 151–198.
18. Kulcsár V, Mérai D. Roman or Barbarian? Provincial models in a Sarmatian pottery center on the Danube frontier. In: De Sena EC, Dobrzańska H, editors. The Roman Empire and beyond: Archaeological and historical research on the Roman and native cultures in Central Europe (BAR International Series 2236). Oxford, UK: Archaeopress; 2011. pp. 61–80.
19. Istvánovits E, Kulcsár V, Mérai D. Roman Age Barbarian pottery workshops in the Great Hungarian Plain. In: Bemmann J, Hegewisch M, Meyer M, Schmauder M, editors. Drehscheibentöpferei im Barbaricum – Technologietransfer und Professionalisierung eines Handwerks am Rande des Römischen Imperiums. Akten der Internationalen Tagung in Bonn vom 11. bis 14. Juni 2009. Bonner Beiträge zur Vor- und Frühgeschichtlichen Archäologie 13. Bonn, Germany: Institut für Vor- und Frühgeschichtliche Archäologie Rheinische Friedrich Wilhelms Universität Bonn; 2011. pp. 355–369.

20. Gabler D, Vaday A. Terra sigillata im Barbaricum zwischen Pannonien und Dazien. (Fontes archaeologici Hungariae). Budapest, Hungary: Akadémiai Kiadó; 1986.
21. Masek Z. Dakische Mode an den pannonischen Limes? Zur Akkulturation der ungarischen Tiefebene in der frührömischen Kaiserzeit. In: Karwowski M, Komoróczy B, Trebsche P, editors. Auf den Spuren der Barbaren: Archäologisch, Historisch, Numismatisch (Archäologie der Barbaren 2015). Brno, Czech Republic: Institute of Archaeology of the Czech Academy of Sciences; 2019. pp. 95–116.
22. Istvánovits E, Kulcsár V. Sarmatians on the borders of the Roman Empire. Steppe traditions and imported cultural phenomena. Anc Civiliz Scythia Sib. 2020;26(2): 391–402. doi: 10.1163/15700577-12341381
23. Kulcsár V. New data on the Germanic–Sarmatian border contacts in Northern Hungary. Acta Musei Porolissensis 1997;21: 705–716.
24. Istvánovits E, Kulcsár V. Iranian-Germanic contacts in the Sarmatian Barbaricum of the Carpathian Basin. In: Mączyńska M, Grabarczyk TW, editors. Die spätrömische Kaiserzeit und die frühe Völkerwanderungszeit in Mittel- und Osteuropa. Łódż, Poland: Wydawnictwo Uniwersytetu Łódzkiego; 2000. pp. 237–260.
25. Istvánovits E, Kulcsár V. Some traces of Sarmatian–Germanic contacts in the Great Hungarian Plain. In: von Carnap-Bornheim C, editor. Kontakt – Kooperation – Konflikt. Germanen und Sarmaten zwischen dem 1. und dem 4. Jahrhundert nach Christus. Internationales Kolloquium des Vorgeschichtlichen Seminars der Philipps-Universität Marburg, 12.−16. Februar 1998. Schriften des Archäologischen Landesmuseums. Band 1. Veröffentlichungen des Vorgeschichtlichen Seminars Marburg, Sonderband 13. Neumünster, Germany: Wachholtz Verlag; 2003. pp. 227–238.
26. Kulcsár V. A kárpát-medencei szarmaták temetkezési szokásai (Múzeumi Füzetek 49). Aszód, Hungary: Osváth Gedeon Museum Foundation; 1998.

27. Grumeza L. Sarmatian cemeteries from Banat (late 1st–early 5th centuries AD). Cluj-Napoca, Romania: Mega Publishing House; 2014.
28. Masek Z. “Barbárok?” – A rákóczifalvi késő szarmata–hun kori pusztulási horizont értékelése ["Barbarians?" – Interpretation of the Late Sarmatian-Hunnic period destruction horizon at Rákóczifalva]. In: Türk A, editor. Hadak Útján XXIV.: A népvándorláskor fiatal kutatóinak XXIV. konferenciája. Esztergom 2014, november 4–6. Budapest & Esztergom, Hungary: Archaeolingua; 2015. pp. 371–406.
29. Vaday A. Atipikus szarmata telepjelenség a Kompolt–Kistéri tanya 15. lelőhelyén. Agria 1997;33: 77–107.
30. Istvánovits E. Tiszavasvári–Városföldje, Jegyző-tag – A settlement of the 5th century. A Nyíregyházi Jósa András Múzeum Évkönyve 1999;41: 173–254.
