# Supplementary material for: White plague among the “forgotten people” from the Barbaricum of the Carpathian Basin–Cases with tuberculosis from the Sarmatian-period (3rd–4th centuries CE) archaeological site of Hódmezővásárhely–Kenyere-ér, Bereczki-tanya (Hungary)
Source: PLoS One. 2024 Jan 10;19(1):e0294762. doi: 10.1371/journal.pone.0294762 (PMC10781108; doi:10.1371/journal.pone.0294762)
Supplement: S2 Text — (PDF) [file pone.0294762.s002.pdf]

S2 Text: Differential diagnoses of the skeletal lesions indicative of tuberculous involvement of the spine that were detected in HK225.

Although spinal tuberculosis (STB) seems to be the most likely underlying cause of the bony changes that were observed in the vertebral column of **HK225**, other aetiologies should also be considered in the differential diagnosis. The most relevant ones are pyogenic spinal infections and granulomatous spinal infections other than TB (fungal infections – e.g., aspergillosis and candidiasis; and bacterial infections – e.g., actinomycosis and brucellosis) [1-5].

Pyogenic spondylitis is the most frequent type of spinal infections, with most cases being diagnosed in patients over 50 years of age [6-9]. It is a rare bacterial infection of the vertebral column, which is caused by pyogenic organisms, most commonly *Staphylococcus aureus* [10-13]. In pyogenic spondylitis, the lumbar region represents the most frequently affected site, followed by the thoracic and cervical spine [6,10,13-14]. The disease typically affects two contiguous vertebrae and the intervening intervertebral disc; unlike in STB, involvement of more than two vertebrae is unusual in pyogenic spondylitis [6-9,15-17]. In most cases, it is the anterior portion of the vertebral body that is primarily affected by the pyogenic infection, whereas involvement of the posterior vertebral elements is fairly rare [7,9]. Vertebral body collapse and consequent kyphosis formation are not typical features of the disease [7]. In contrast to STB, pyogenic spondylitis is characterised by rapid, massive bone regeneration (e.g., sclerosis, reactive new bone formations, and osteophyte-like bony extensions) but formation of extra-vertebral abscesses and sub-ligamentous spread of the infection are less frequent [9-11,15,17-19]. Based on the young age of **HK225**, as well as the presence of vertebral body collapse, the evident signs of overlying extra-vertebral abscesses and sub-ligamentous spread of the infection, and the absence of sclerotic repair of the lytic lesion in the spine of **HK225**, pyogenic spondylitis can be ruled out with high certainty as a diagnostic option in **HK225.**

Although in recent years, the incidence of fungal granulomatous infections has been increased, they are still extremely rare, with candidiasis and aspergillosis being the most frequent ones with potential spinal involvement [11,20-25]. They are caused by *Candida* and *Aspergillus* species, respectively [11,21,25]. In fungal spondylitis, it is the lumbar region, which is the most commonly involved site, followed by the thoracic and cervical regions [20,22-23,25-26]. Similar to STB, fungal spondylitis can affect not only one but multiple vertebrae, with multi-level non-contiguous involvement being not unusual [27-28]. It can cause vertebral body collapse and consequent kyphosis formation [29]. Furthermore, fungal spondylitis is often associated with the formation of extra-vertebral abscesses and sub-ligamentous spread of the infection, and unlike STB, it frequently affects the posterior vertebral elements [27-31]. Based on the rarity of fungal spondylitis, as well as the absence of posterior vertebral element involvement, it is unlikely that it was fungal spondylitis that resulted in the development of the skeletal lesions that were observed in the vertebral column of **HK225**.

Although actinomycosis, a rare bacterial granulomatous infection caused by *Actinomyces* species, can result in vertebral osteomyelitis, actinomycotic involvement of the spine is extremely rare and mainly occurs in adults [29,32-36]. In most cases with actinomycotic spondylitis, the disease affects the cervical and thoracic regions of the vertebral column [33-36]. Usually not one but several contiguous vertebrae are involved, and there is a tendency for the posterior vertebral elements to be affected rather than the vertebral bodies; thus, actinomycotic spondylitis scarcely leads to vertebral body collapse and consequent kyphosis formation [29,33,37-39]. The disease is characterised by formation of extra-vertebral abscesses and sub-ligamentous spread of the infection, which can lead to the formation of cortical erosions, sclerosis, and reactive new bone formations; osteolytic lesions and osteophyte-like bony extensions can also occur [29,33-34,37-39]. Based on the age preference and rarity of actinomycotic spondylitis, as well as the presence of vertebral body collapse and the absence

of posterior vertebral element involvement in **HK225**, it is unlikely that an infection with *Actinomyces* spp. resulted in the development of the bony changes that were detected in the vertebral column of **HK225**.

Brucellosis is a bacterial granulomatous infection caused by *Brucella* species, which can spread from animals to humans [29,40-43]. Spinal involvement, i.e., brucellar spondylitis, mainly occurs in adults, especially those over 50 years of age [29,40,43-45]. It is the lumbar region of the vertebral column that is most commonly affected by the disease, followed by the thoracic and cervical spine; usually two contiguous vertebral bodies are involved [40-41,43,45-46]. Unlike in STB, vertebral body collapse and consequent kyphosis formation, as well as development of extra-vertebral abscesses are not typical features of brucellar spondylitis. In cases where there is extra-vertebral abscess formation, they tend to be small, relatively limited, and usually do not spread [29,42,44-45,47-48]. Brucellar spondyilitis is characterised by concomitant occurrence of reparative and destructive processes, with sclerosis, reactive new bone formations, and osteophyte-like bony extensions [29,40,44-47]. Based on the young age of **HK225**, as well as the presence of vertebral body collapse, the evident signs of overlying extra-vertebral abscesses and sub-ligamentous spread of the infection, and the absence of sclerotic repair of the lytic lesion in the spine of **HK225**, brucellosis seems to be less likely to be responsible for the development of the skeletal lesions that were observed in the vertebral column of **HK225**.

REFERENCES:

1. Garg RK, Somvanshi DS. Spinal tuberculosis: A review. J Spinal Cord Med. 2011;34(5): 440–454. doi: 10.1179/2045772311Y.0000000023

2. Agrawal A, Timothy J, Shetty MS, Shetty L, Shetty JP. Paediatric perspective of spinal tuberculosis. Paediatr Today 2006;9(1): 25−34.

3. Matos V, Marques C, Lopes C. Severe vertebral collapse in a juvenile from the graveyard (13th/14th–19th centuries) of the São Miguel Church (Castelo Branco, Portugal): Differential palaeopathological diagnosis. Int J Osteoarchaeol. 2011;21(2): 208−217. doi: 10.1002/oa.1125
4. Sparacello VS, Roberts CA, Kerudin A, Müller R. A 6500-year-old Middle Neolithic child from Pollera Cave (Liguria, Italy) with probable multifocal osteoarticular tuberculosis. Int J Paleopathol. 2017;17: 67−74. doi: 10.1016/j.ijpp.2017.01.004
5. Dean A, Zyck S, Toshkezi G, Galgano M, Marawar S. Challenges in the diagnosis and management of spinal tuberculosis: case series. Cureus 2019;11(1): e3855. doi: 10.7759/cureus.3855
6. Cheung WY, Luk KDK. Pyogenic spondylitis. Int Orthop. 2012;36(2): 397–404. doi: 10.1007/s00264-011-1384-6
7. Lee KY. Comparison of pyogenic spondylitis and tuberculous spondylitis. Asian Spine J. 2014;8(2): 216−223. doi: 10.4184/asj.2014.8.2.216
8. Nickerson EK, Sinha R. Vertebral osteomyelitis in adults: An update. Br Med Bull. 2016;117(1): 121−138. doi: 10.1093/bmb/ldw003
9. Sato K, Yamada K, Yokosuka K, Yoshida T, Goto M, Matsubara T, et al. Pyogenic spondylitis: Clinical features, diagnosis and treatment. Kurume Med J. 2019;65(3): 83–89. doi: 10.2739/kurumemedj.MS653001
10. Hasan NMA. Pedicle involvement in tuberculous spondylitis and pyogenic spondylitis: Comparative magnetic resonance imaging study. Egypt J Radiol Nucl Med. 2014;45(2): 455–460. doi: 10.1016/j.ejrnm.2014.02.011
11. Acharya J, Gibbs WN. Imaging spinal infection. Radiol Infect Dis. 2016;3(2): 84–91. doi: 10.1016/j.jrid.2016.03.001

12. Kwon J-W, Hyun S-J, Han S-H, Kim K-J, Jahng T-A. Pyogenic vertebral osteomyelitis: Clinical features, diagnosis, and treatment. Korean J Spine 2017;14(2): 27–34. doi: 10.14245/kjs.2017.14.2.27

13. Sakti YM, Chusnanto AR, Resubun AP, Putro AC, Cein CR, Tampubolon YO, et al. Novel minimally invasive management of lumbar osteomyelitis: A case report. Int J Surg Case Rep. 2022;92: 106878. doi: 10.1016/j.ijscr.2022.106878

14. Skaf GS, Domloj NT, Fehlings MG, Bouclaous CH, Sabbagh AS, Kanafani ZA, et al. Pyogenic spondylodiscitis: An overview. J Infect Public Health 2010;3(1): 5–16. doi: 10.1016/j.jiph.2010.01.001

15. Alvik I. Chronic pyogenic spondylitis – Tuberculous spondylitis. Acta Orthop Scand. 1951;21(2–4): 237–242. doi: 10.3109/17453675109024158

16. Mavrogenis AF, Megaloikonomos PD, Igoumenou VG, Panagopoulos GN, Giannitsioti E, Papadopoulos A, et al. Spondylodiscitis revisited. EFORT Open Rev. 2017;2(11): 447–461. doi: 10.1302/2058-5241.2.160062

17. Yueniwati Y, Christina E. The challenges in differentiating tuberculous from pyogenic spondylitis using magnetic resonance imaging. Rep Medical Imaging 2017;10: 37–43. doi: 10.2147/RMI.S129533

18. Park J-H, Shin H-S, Park JT, Kim TY, Eom KS. Differentiation between tuberculous spondylitis and pyogenic spondylitis on MR imaging. Korean J Spine 2011;8(4): 283–287. doi: 10.14245/kjs.2011.8.4.283

19. Rivas-Garcia A, Sarria-Estrada S, Torrents-Odin C, Casas-Gomila L, Franquet E. Imaging findings of Pott's disease. Eur Spine J. 2013;22(Suppl. 4): S567–S578. doi: 10.1007/s00586-012-2333-9

20. Kim CW, Perry A, Currier B, Yaszemski M, Garfin SR. Fungal infections of the spine. Clin Orthop Relat Res. 2006;444: 92−99. doi: 10.1097/01.blo.0000203451.36522.4c

21. Sethi S, Siraj F, Kalra KL, Chopra P. *Aspergillus* vertebral osteomyelitis in immunocompetent patients. Indian J Orthop. 2012;46(2): 246–250. doi: 10.4103/0019-5413.93693

22. Lee S-W, Lee SH, Chung HW, Kim MJ, Seo MJ, Shin MJ. *Candida* spondylitis: Comparison of MRI findings with bacterial and tuberculous cases. Am J Roentgenol. 2013;201(4): 872−877. doi: 10.2214/AJR.12.10344

23. Caldera G, Cahueque M, Cobar A, Gómez G, Rodríguez O. Fungal spondylodiscitis: Review. J Spine 2016;5(2): 1000302. doi: 10.4172/2165-7939.1000302

24. Panyaping T. Imaging of infectious spondylitis. Rama Med J. 2017;40(1): 51–66.

25. Dai G, Wang T, Yin C, Sun Y, Xu D, Wang Z, et al. *Aspergillus* spondylitis: Case series and literature review. BMC Musculoskelet Disord. 2020;21: 572. doi: 10.1186/s12891-020-03582-x

26. Oh I-S, Seo J-Y, Ha K-Y, Kim Y-C. Treatment for multiple *Aspergillus* spondylitis including a hip joint. Asian Spine J. 2009;3(2): 106−112. doi: 10.4184/asj.2009.3.2.106

27. Park SW, Jeong JH, Choi S-H, Kim YS, Woo JH, Jeon SR. Candidal spondylitis: Five new cases and a review of previously reported cases. Korean J Spine 2007;4(1): 31–36.

28. Kwon JW, Hong SH, Choi S-H, Yoon YC, Lee SH. MRI findings of *Aspergillus* spondylitis. Am J Roentgenol. 2011;197(5): W919–W923. doi: 10.2214/AJR.11.6786

29. Aufderheide AC, Rodríguez-Martín C. The Cambridge encyclopedia of human paleopathology. Cambridge, UK: Cambridge University Press; 1998.

30. Ortner DJ. Infectious diseases: Tuberculosis and leprosy. In: Ortner DJ, editor. Identification of pathological conditions in human skeletal remains. San Diego, CA, USA: Academic Press; 2003. pp. 227–271.

31. Moorthy S, Prabhu NK. Spectrum of MR imaging findings in spinal tuberculosis. Am J Roentgenol. 2002;179(4): 979–983. doi: 10.2214/ajr.179.4.1790979

32. Honda H, Bankowski MJ, Kajioka EHN, Chokrungvaranon N, Kim W, Gallacher ST. Thoracic vertebral actinomycosis: *Actinomyces israelii* and *Fusobacterium nucleatum*. J Clin Microbiol. 2008;46(6): 2009−2014. doi: 10.1128/JCM.01706-07
33. Duvignaud A, Ribeiro E, Moynet D, Longy-Boursier M, Malvy D. Cervical spondylitis and spinal abscess due to *Actinomyces meyeri*. Braz J Infect Dis. 2014;18(1): 106–109. doi: 10.1016/j.bjid.2013.05.016
34. Patil VR, Joshi AR, Joshi SS, Patel D. Lumbosacral actinomycosis in an immunocompetent individual: An extremely rare case. J Craniovertebr Junction Spine 2014;5(4): 173–175. doi: 10.4103/0974-8237.147088
35. Ronceray L, Friesenbichler W, Hutter C, Lakatos K, Krizmanich W, Amann G, et al. Thoracic actinomycosis with infiltration of the spine: An oncological pitfall. J Pediatr Hematol Oncol. 2018;40(6): 468–471. doi: 10.1097/MPH.0000000000001035
36. Yamada Y, Kinoshita C, Nakagawa H. Lumbar vertebral osteomyelitis and psoas abscess caused by *Actinomyces isreaelii* after an operation under general anesthesia in a patient with end-stage renal disease: A case report. J Med Case Rep. 2019;13: 151. doi: 10.1186/s13256-019-2261-y
37. Cope VZ. Actinomycosis of bone with special reference to infection of the vertebral column. J Bone Joint Surg. 1951;33B(2): 205–214.
38. Ernst J, Ratjen E. Actinomycosis of the spine: Report of two cases. Acta Orthop Scand. 1971;42(1): 35–44. doi: 10.3109/17453677108989023
39. Hadgaonkar S, Rathi P, Vincent V, Shyam A, Sancheti P. Actinomycotic infection of spine – A rare disease with diagnostic challenge, an update on spinal infection. J Orthop Case Rep. 2021;11(1): 72–78. doi: 10.13107/jocr.2021.v11.i01.1970
40. Bodur H, Erbay A, Colpan A, Akinci E. Brucellar spondylitis. Rheumatol Int. 2004;24(4): 221–226. doi: 10.1007/s00296-003-0350-z

41. Lee HJ, Hur JW, Lee JW, Lee SR. Brucellar spondylitis. J Korean Neurosurg Soc. 2008;44(4): 277–279. doi : 10.3340/jkns.2008.44.4.277
42. Li T, Li W, Du Y, Gao M, Liu X, Wang G, et al. Discrimination of pyogenic spondylitis from brucellar spondylitis on MRI. Medicine 2018;97(26): e11195. doi: 10.1097/MD.0000000000011195
43. Zhang Y, Zhang Q, Zhao C. Cervical brucellar spondylitis causing incomplete limb paralysis. Rev Soc Bras Med Trop. 2019;52: e20180243. doi: 10.1590/0037-8682-0243-2018
44. Ranjbar M, Turgut AT, Nojomi M, Turgut M. Brucellar spondylitis as a complication of brucellosis. In: Turgut M, Haddad FS, de Divitiis O, editors. Neurobrucellosis. Clinical, diagnostic and therapeutic features. Cham, Switzerland: Springer; 2015. pp. 89−98. doi: 10.1007/978-3-319-24639-0
45. Shen L, Jiang C, Jiang R, Fang W, Feng Q, Wang L, et al. Diagnosis and classification in MRI of brucellar spondylitis. Radiol Infect Dis. 2017;4(3): 102–107. doi: 10.1016/j.jrid.2017.08.005
46. Yang B, Hu H, Chen J, He X, Li H. The evaluation of the clinical, laboratory, and radiological findings of 16 cases of brucellar spondylitis. Biomed Res Int. 2016;2016: 8903635. doi: 10.1155/2016/8903635
47. Ortner DJ. Infectious diseases: Introduction, biology, osteomyelitis, periostitis, brucellosis, glanders, and septic arthritis. In: Ortner DJ, editor. Identification of pathological conditions in human skeletal remains. San Diego, CA, USA: Academic Press; 2003. pp. 179–226.
48. Guo H, Lan S, He Y, Tiheiran M, Liu W. Differentiating *Brucella* spondylitis from tuberculous spondylitis by the conventional MRI and MR T2 mapping: A prospective study. Eur J Med Res. 2021;26(1): 125. doi: 10.1186/s40001-021-00598-4
