# Supplementary material for: White plague among the “forgotten people” from the Barbaricum of the Carpathian Basin–Cases with tuberculosis from the Sarmatian-period (3rd–4th centuries CE) archaeological site of Hódmezővásárhely–Kenyere-ér, Bereczki-tanya (Hungary)
Source: PLoS One. 2024 Jan 10;19(1):e0294762. doi: 10.1371/journal.pone.0294762 (PMC10781108; doi:10.1371/journal.pone.0294762)
Supplement: S3 Text — (PDF) [file pone.0294762.s003.pdf]

S3 Text: Differential diagnoses of the bony changes indicative of tuberculous involvement of both hip joints that were observed in HK225.

Although TB arthritis seems to be the most likely underlying cause of the skeletal lesions (localised peri-articular osteoporosis) that were detected in both hip joints of **HK225**, other aetiologies should also be considered in the differential diagnosis. The most relevant ones are rheumatoid arthritis and pyogenic arthritis [1-5].

Rheumatoid arthritis (RA) is a chronic, systemic autoimmune disease of the joints, with a typical onset occurring in adults in their fourth or fifth decade of life [6-12]. RA initially targets the synovial lining of the peripheral joints; the disease tends to concomitantly affect multiple sites in a bilaterally symmetrical distribution [6-8,11]. Although any synovial joint can become involved in RA, it usually commences in the small joints of the hands and feet, and later can progress to the larger joints of the limbs (e.g., shoulder, hip, elbow or knee) [6-8,10-12]. Based on the age preference of RA, and the absence of signs of arthritis in the small joints of the hands and feet of **HK225**, RA seems to be less likely to be responsible for the development of the bony changes (localised peri-articular osteoporosis) that were observed in the hip joints of **HK225**.

Pyogenic arthritis is a rapidly progressive joint disease that is caused by aggressive pyogenic bacteria, predominantly *Staphylococcus* and *Streptococcus* spp. [7,13-17]. In most cases, it is an acute mono-arthritis, with the knee and hip joints representing the most common sites of involvement [7,13,17]. Unlike in TB arthritis, the pathological process is rapid and bone destruction is usually much less extensive in pyogenic arthritis [7,13,17]. As in dry bone, the initial (‘synovitis’) stage of the disease is very similar to that of TB arthritis, differentiation from one another may be impossible [13,17]; thus, pyogenic arthritis cannot be excluded in the differential diagnosis of the localised peri-articular osteoporosis that was observed in both hip

joints of **HK225**. Nonetheless, its concomitant occurrence with other bony changes indicative of osteoarticular TB, as well as the tuberculous involvement of the meninges of **HK225**, which provides evidence that they suffered from TB at the time of death, makes TB arthritis a more likely underlying cause.

REFERENCES:

1. Hong SH, Kim SM, Ahn JM, Chung HW, Shin MJ, Kang HS. Tuberculous versus pyogenic arthritis: MR Imaging evaluation. Radiology 2001;218(3): 848–853. doi: 10.1148/radiology.218.3.r01fe27848é
2. Choi J-A, Koh SH, Hong S-H, Koh YH, Choi J-Y, Kang HS. Rheumatoid arthritis and tuberculous arthritis: Differentiating MRI features. Am J Roentgenol. 2009;193(5): 1347–1353. doi: 10.2214/AJR.08.2164
3. Chen C, Yin Y, Xu H, Chen G. Early clinical outcomes of one-stage total hip arthroplasty for the treatment of advanced hip tuberculosis. J Orthop Surg (Hong Kong) 2021;29(1): 23094990211000143. doi: 10.1177/23094990211000143
4. Goodall JW, Patterson BJ, Barrett J, Colquhoun M, Williamson S, Clayton-Smith A, et al. A comparison of tuberculous and bacterial native joint septic arthritis infections in a retrospective cohort: Presentation characteristics, outcomes and long term follow up. Clin Infect Pract. 2022;13: 100138. doi: 10.1016/j.clinpr.2022.100138
5. Jung Y, Choi BY. Differential diagnosis of inflammatory arthropathy accompanying active tuberculosis infection. J Rheum Dis. 2022;29(2): 108–115. doi: 10.4078/jrd.2022.29.2.108
6. Ortner DJ, Putschar WGJ. Identification of pathological conditions in human skeletal remains. Washington, DC, USA: Smithsonian Institution Press; 1981. pp. 403-411.
7. Aufderheide AC, Rodríguez-Martín C. The Cambridge encyclopedia of human paleopathology. Cambridge, UK: Cambridge University Press; 1998.

8. Ortner DJ. Erosive arthropathies, enthesopathies, and miscellaneous pathological conditions of joints. In: Ortner DJ, editor. Identification of pathological conditions in human skeletal remains. San Diego, CA, USA: Academic Press; 2003. pp. 561−587.
9. Guo Q, Wang Y, Xu D, Nossent J, Pavlos NJ, Xu J. Rheumatoid arthritis: Pathological mechanisms and modern pharmacologic therapies. Bone Res. 2018;6: 15. doi: 10.1038/s41413-018-0016-9
10. Smolen JS, Aletaha D, Barton A, Burmester GR, Emery P, Firestein GS, et al. Rheumatoid arthritis. Nat Rev Dis Primers 2018;4: 18001. doi: 10.1038/nrdp.2018.1
11. Bullock J, Rizvi SAA, Saleh AM, Ahmed SS, Do DP, Ansari RA, et al. Rheumatoid arthritis: A brief overview of the treatment. Med Princ Pract. 2019;27(6): 501-507. doi: 10.1159/000493390
12. Boldeanu MV, Ionescu AR, Popoviciu VH, Bărbulescu AL, Dinescu ŞC, Siloşi I, et al. Diagnostic challenges and management update in rheumatoid arthritis. In: Mohammed RHA, editor. Rheumatoid arthritis – Other perspectives towards a better practice. London, UK: IntechOpen; 2020. pp. 294-427. doi: 10.5772/intechopen.91965
13. Ortner DJ. Infectious diseases: Introduction, biology, osteomyelitis, periostitis, brucellosis, glanders, and septic arthritis. In: Ortner DJ, editor. Identification of pathological conditions in human skeletal remains. San Diego, CA, USA: Academic Press; 2003. pp. 179–226.
14. Sakurai A, Okahashi N, Nakagawa I, Kawabata S, Amano A, Ooshima T, et al. *Streptococcus pyogenes* infection induces septic arthritis with increased production of the receptor activator of the NF-κB ligand. Infect Immun. 2003;71(10): 6019–6026. doi: 10.1128/IAI.71.10.6019-6026.2003
15. Dubost J-J, Soubrier M, De Champs C, Ristori J-M, Sauvezie B. Streptococcal septic arthritis in adults. A study of 55 cases with a literature review. Joint Bone Spine 2004;71(4): 303–311. doi: 10.1016/S1297-319X(03)00122-2

16. Berendt AR. Pyogenic arthritis. In: Warrell DA, Cox TM, Firth JD, editors. Oxford textbook of medicine. Oxford; 2010. doi: 10.1093/med/9780199204854.003.1907
17. Roberts CA. Infectious disease: Introduction, periostosis, periostitis, osteomyelitis, and septic arthritis. In: Buikstra JE, editor. Ortner's Identification of pathological conditions in human skeletal remains, San Diego, CA, USA: Academic Press; 2019, pp. 285–319. doi: 10.1016/B978-0-12-809738-0.00011-9
