# Supplementary material for: White plague among the “forgotten people” from the Barbaricum of the Carpathian Basin–Cases with tuberculosis from the Sarmatian-period (3rd–4th centuries CE) archaeological site of Hódmezővásárhely–Kenyere-ér, Bereczki-tanya (Hungary)
Source: PLoS One. 2024 Jan 10;19(1):e0294762. doi: 10.1371/journal.pone.0294762 (PMC10781108; doi:10.1371/journal.pone.0294762)
Supplement: Attachment — Submitted filename: response_to_reviewers.docx [file pone.0294762.s005.docx]

Dr Dario Piombino-Mascali, PhD

Academic Editor

PLOS ONE

Dear Dr Piombino-Mascali,

We are very thankful for your comments regarding our manuscript entitled “*White Plague among the “Forgotten People” from the Barbaricum of the Carpathian Basin – Cases with tuberculosis from the Sarmatian-period (3^rd^–4^th^ centuries CE) archaeological site of Hódmezővásárhely–Kenyere-ér, Bereczki-tanya (Hungary)*” that was submitted to PLOS ONE (manuscript ID: PONE-D-23-20370). The main text has been modified following your suggestions and was copyedited for language usage, spelling, and grammar by Dr David R. Hunt, PhD.

The revised manuscript file has been uploaded to the submission site of the journal. We hope this new version will be suitable for publication in PLOS ONE.

Thank you again for your comments and your editorial work!

Yours sincerely,

Dr Olga Spekker, PhD

Corresponding author

University of Szeged, Közép fasor 52, H-6726, Szeged, Hungary

Email: [olga.spekker@gmail.com](mailto:olga.spekker@gmail.com)Tel: +36 20 807 72 94
